# Down-Regulation of PDCD4 Promotes Proliferation, Angiogenesis and Tumorigenesis in Glioma Cells

**DOI:** 10.3389/fcell.2020.593685

**Published:** 2020-11-12

**Authors:** Guo Pin, Li Huanting, Zhu Chengzhan, Kong Xinjuan, Feng Yugong, Liu Wei, Li Shifang, Li Zhaojian, Han Kun, Yao Weicheng, Lin Yingying, Qiu Yongming, Yu Yanan

**Affiliations:** ^1^Department of Neurosurgery, The Affiliated Hospital of Qingdao University, Qingdao, China; ^2^Institute of Cerebral Vascular Diseases, The Affiliated Hospital of Qingdao University, Qingdao, China; ^3^Department of Hepatobiliary and Pancreatic Surgery, The Affiliated Hospital of Qingdao University, Qingdao, China; ^4^Department of Gastroenterology, The Affiliated Hospital of Qingdao University, Qingdao, China; ^5^Department of Neurosurgery, Renji Hospital, School of Medicine, Shanghai Jiao Tong University, Shanghai, China

**Keywords:** glioma, PDCD4, AKT, STAT3, angiogenesis

## Abstract

The programmed cell death 4 (*PDCD4*) tumor-suppressor gene regulates cell apoptosis, protein translation, signal transduction, and induction of mediators of inflammation. However, the mechanism by which *PDCD4* is down-regulated and regulates tumor growth remains elusive. In this study, we showed that PDCD4 is down-regulated in glioma cells and acts as a tumor suppressor. Based on the TCGA data, we confirmed that AKT2, but not AKT1 or AKT3, interacts with PDCD4, thus leading to the suppression of PDCD4 in glioma cells. Moreover, the analysis suggested that PDCD4 regulates the expression of IL-5, CCL-5, VEGF, and CXCL10 via the NF-kB pathway. Additionally, depletion of levels of *PDCD4* promoted angiogenic activity of glioma cells via the VEGF-STAT3 pathway. When tumor cells over-expressing *PDCD4* were injected into nude mice, the increased expression of PDCD4 blocked tumorigenesis and prolonged overall survival. Our study indicates the need to develop drugs that can modulate the expression of PDCD4 and test their efficacy in clinical trials.

## Introduction

Glioblastoma multiforme is the most aggressive tumor of the central nervous system, with an annual incidence rate of approximately 6 per 100,000 individuals (Hanif et al., 2017). The initiation and progression of glioma is regulated by various factors, including copy number variations, somatic mutations, epigenetic modifications, metabolic conversion, stem cell pathways, gene fusions and tumor microenvironment (Gladson et al., 2010). Despite the combination of surgery, chemotherapy and radiotherapy, the prognosis of the patients remains poor due to its molecular heterogeneity and high degree of invasiveness (Davis, 2016). Therefore, it is imperative to further explore efficient molecular-targeted therapies of glioma.

The human programmed cell death 4 (PDCD4) was considered to encode a nuclear antigen and located on chromosome 10 (Azzoni et al., 1998). The expression of PDCD4 is regulated by interleukins IL-2, IL-12, and IL-15 (Ozpolat et al., 2007). The PDCD4 acts as a tumor-suppressor gene and performs essential functions in many biological events, including apoptosis, protein translation, signal transduction, and stimulation of inflammattion mediators (Zhang et al., 2014). Cmarik et al. (1999) reported that expression of PDCD4 is less in the mouse JB6 promotion-sensitive cells than that in promotion-resistant cells, and it functions as an inhibitor of neoplastic transformation. The PDCD4 binds to eIF4A and inhibits its helicase activity, and suppresses protein synthesis (Wang and Yang, 2018). A previous study reported that PDCD4 associates with several transcription factors and regulates transcription. For example, PDCD4 directly interacts with the transcription factor TWIST1, which inhibits target genes downstream to TWIST1 and thus results in the inhibition of cell proliferation (Shiota et al., 2009). Additionally, PDCD4 also interacts with the cytoplasmic factors, such as PABP and DAXX (Shiota et al., 2009).

The expression of PDCD4 is tightly regulated at the level of transcription, translation, and protein degradation (Matsuhashi et al., 2019). Leupold et al. (2012) reported that transcription of PDCD4 is regulated by zinc-finger protein transcription factors such as specificity protein (SP) and ZBP-89. Moreover, the translation of the *PDCD4* mRNA is inhibited by several microRNAs, including microRNA-21, microRNA-182, microRNA-16, microRNA-150, and microRNA-499 (Matsuhashi et al., 2019). Further, Dorrello et al. reported that the PDCD4 is phosphorylated by the ribosomal protein S6 kinase 1, PI3K pathway, and mTOR signaling pathway, and is then ubiquitinated and degraded in proteasomes (Matsuhashi et al., 2019). Additionally, MEK-ERK signaling pathway facilitated substantial degradation of PDCD4 via proteasomes.

The loss of expression of *PDCD4* is diagnostic indicator for different human cancers, and is prognostic indicator for survival in cancers of the breast, liver, colon, lung, glioma, and esophagus (Lankat-Buttgereit and Goke, 2009). Moreover, the inhibition or deficiency of PDCD4 enhances tumorigenesis and tumor progression *in vivo*. The over-expression of *PDCD4* decreases anchorage-independent growth *in vitro*, and prevents tumor growth in the xenograft mouse model (Matsuhashi et al., 2019). Furthermore, gene therapy by targeting *PDCD4* in an activated K-Ras model was shown to block the development of lung cancer (Wang et al., 2016). However, the functions of PDCD4 and the mechanism by which *PDCD4* is down-regulated in human glioma cells remains to be completely elucidated.

## Materials and Methods

### Cell Culture and Reagents

All human cell lines were obtained from the American Type Culture Collection (ATCC), which performs authentication on its own cell lines. All the cell lines were cultured in DMEM supplemented with 10% heat-inactivated FBS at 37°C and 5% CO_2_ in a humidified incubator. Antibodies against total PDCD4 (CAT#:9535S), AKT1 (CAT#:75692), AKT2 (CAT#:3063), AKT3 (CAT#:14982), P65 (CAT#:8242), P50 (CAT#:3035), c-Rel (CAT#:4727), RelB (CAT#:10544), Tubulin (CAT#:2148s), Histone3 (CAT#:4499), Actin (CAT#:3700), STAT3 (CAT#:9139), ERK(CAT#:4695), FAK1 (CAT#:3285), phospho-STAT3 (CAT#:9145), phospho-ERK (CAT#:4370), and phospho-FAK1 (CAT#:8556) were purchased from Cell Signaling Technology. Antibody against VEGF (CAT#:ab52917) was purchased from Abcam. Human Cytokine Antibody Array C3 was purchased from Raybio.

### Ethics Statement

The laboratory and clinical protocols of this study were approved by the Ethics Committee and Clinical Research Board at the Affiliated Hospital of Qingdao University (#170540091), Shangdong Province, China.

### Lentivirus-Mediated shRNA Knockdown and Over-Expression of Candidate Genes

Specific shRNA sequences targeting PDCD4, AKT1, AKT2, or AKT3 were inserted into the pGIPZ lentiviral vector (Open Biosystems). The target sequences were as follows: shPDCD4-1: CACCAATCATACAGGAATA, shPDCD4-2: GCTTCTTTCT GACCTTTGT, shAKT1-1: CCATAGTTGCGGGCCCGGTCC, shAKT1-2: AGTGCCCTTGCCCAGCAGC, shAKT2-1: CCAAT GAAGGAGCCGTCGCTC, shAKT2-2: GGGTGGCAGGAGCT TCTTC, shAKT3-1: AGAAACGTGTGCGGTCC, and shAKT3-2: GCTTCTGTCCATTCTTCCC. The PDCD4 coding sequence was cloned into the pHR-SIN lentiviral vector under CMV promoter control for over-expression studies.

### Immunoblotting

The cultured cells were harvested in RIPA lysis buffer (25 mM Tris pH7.4, 150 mM NaCl, 5 mM EDTA, 1% Triton-X, 1_μg/ml pepstatin A, 1 μg/ml leupeptin, 1.5 μg/ml aprotinin, and 0.1 mM phenylmethylsulfonyl fluoride). Equal amounts of denatured protein lysates were resolved in SDS-polyacrylamide gels for electrophoresis and then transferred to polyvinylidene difluoride membranes. The membranes were blocked with 5% non-fat milk in TBS containing 0.1% Tween for 1 h at room temperature, and subsequently probed with primary antibodies overnight at 4°C. The blots were incubated with HRP-conjugated secondary antibodies for 1 h at room temperature, and visualized using enhanced chemiluminescence on the ChemiDoc XRS system.

### Quantitative Real-Time PCR

Total RNA was extracted from cultured cells using the Purelink RNA mini Kit following the manufacturer’s instructions. The qScript cDNA Supermix was used to reverse transcribe 1 μg of total RNA into first strand cDNA. Quantitative real-time PCR was performed using the SYBR-Green Master Mix. Relative gene expression was calculated using the comparative Ct method, which was normalized to the expression of *ACTB* (endogenous control). The specificity of the PCR products was confirmed by analyzing the melting curve. All amplification reactions were performed in triplicates. The primer sequences used to amplify indicated mRNA were as follows: CXCL5 sense, 5′-TGGACGGTGGAAACAAGG-3′; CXCL5 antisense, 5′-CTTCCCTGGGTTCAGAGAC-3′; GAPDH sense, 5′-CGCTCTCTGCTCCTCCTGTTCG-3′; and GAPDH antisense, 5′-CGGCTGGCGACGCAAAAGAAG-3′; CCL5 sense, 5′-ATCCTCATTGCTACTGCCCTC-3′; and CCL5 antisense, 5′-GCCACTGGTGTAGAAATACTCC-3′; CXCL10 sense, 5′-GAACTGTACGCTGTACCTGCA-3′; and CXCL10 antisense, 5′-TTGATGGCCTTCGATTCTGGA-3′; VEGF sense, 5′-TGCAGATTATGCGGATCAAACC-3′; and VEGF antisense, 5′-TGCATTCACATTTGTTGTGCTGTAG-3′; MIP3 sense, 5′-TCCTGGCTGCTTTGATGTCA-3′; and MIP3 antisense 5′-GAAGAATACGGTCTGTGTATCCAAGAC-3′.

### Immunofluorescence

Xenograft tumor samples were sectioned at thickness of 6 μm, air-dried, fixed with ice-cold 4% paraformaldehyde for 15 min, and washed thrice with phosphate-buffered saline (PBS). Sections were permeabilized using 0.1% Triton X-100 in PBS for 15 min at room temperature. After washing thrice with PBS, the slides were blocked using 1% BSA in PBS for 1 h at room temperature. Tissues were incubated with the indicated antibodies overnight in a humidified chamber at 4°C. After washing thrice with PBS containing 1% Tween-20, the slides were incubated with fluorophore-conjugated anti-mouse/-rabbit secondary antibodies and DAPI. Stained slides were observed using the Nikon Eclipse Ti confocal microscope.

### Cytokine Array Analysis

Serum-free DMEM was collected from 90% confluent cancer fibroblasts with stable knockdown of *IRAK4* or scrambled vector, incubated with the membranes from Human Cytokine Antibody Array C3 kit (RayBiotech, Cat#:AAH-CYT-3-2), and then processed according to the manufacturer’s instructions (Zhang et al., 2017, 2018).

### NF-kB Reporter Assay

NF-kB reporter assay was performed with pNL3.2.NF-kB-RE (Promega, catalog number: N1111) using the Dual-Glo Luciferase Assay system. All experiments were performed three times in triplicates, and data are represented as mean ± SEM.

### Colony Formation Assay

The indicated cells were counted and seeded in 6-well plates at density of 500 cells per well. After 14 days of culture, the cell colonies were fixed with 4% paraformaldehyde for 10 min, and subsequently stained with 0.5% crystal violet for 30 min. The cell colonies were counted and photographed.

### Proximal Ligation Assay

The proximal ligation assay was performed using the Duolink *in situ* red starter kit (DUO92101, Sigma-Aldrich) according to the manufacturer’s instructions. The number of puncta per field was recorded using a Nikon C2 + fluorescent confocal microscope.

### Xenograft

For developing xenograft model, 2 × 10^6^ cells of the indicated cell lines were mixed in Matrigel, and subcutaneously injected into the flanks of immunocompromised 8-week-old BALB/c nude mice. Tumor growth was monitored every 2–3 days and represented as tumor area, along with animal weight.

### Statistics

All results, when applicable, were expressed as the mean ± SEM. Statistical analysis was performed using the Prism 6 software program. Unpaired Student’s *t*-test was used to compare two groups when appropriate. *P* < 0.05 were considered to be statistically significant.

## Results

### PDCD4 Is Down-Regulated in Glioma

**FIGURE 1:**
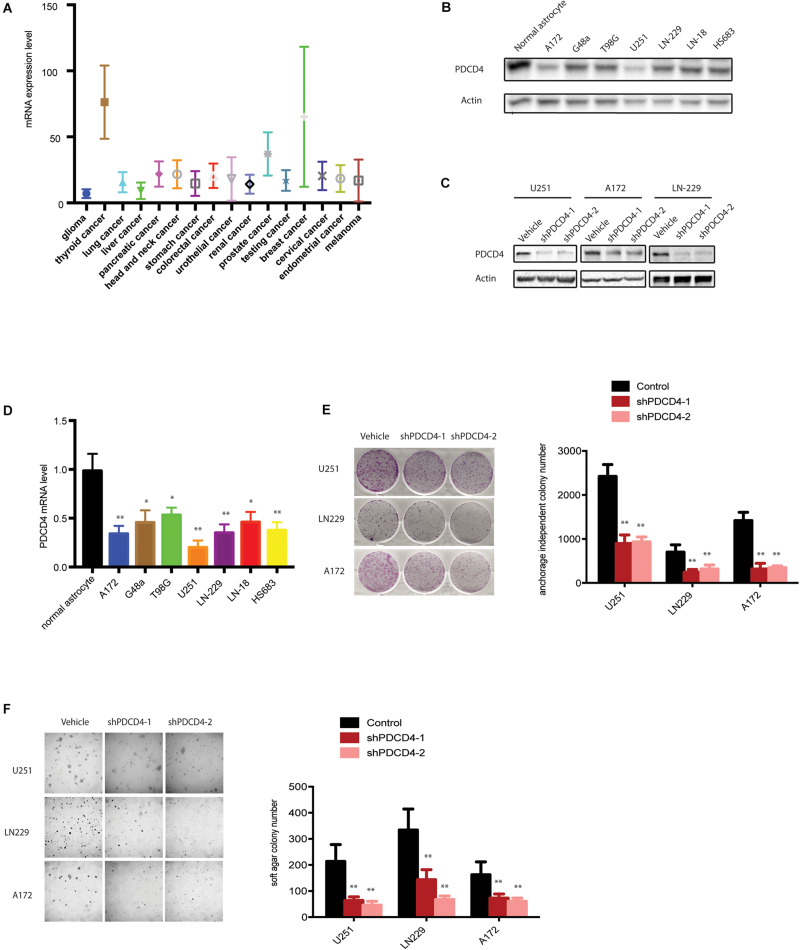
PDCD4 is down-regulated in glioma cells and acts as a tumor suppressor. **(A)** Analysis of mRNA levels in 16 cancer types based on the TCGA dataset. **(B)** Western blot analysis showing PDCD4 protein levels in seven glioma cell lines vs. normal astrocytes. **(C)** Western blot analysis indicating change in PDCD4 protein levels in stable *PDCD4*-knockdown cells. **(D)** Real-time PCR analysis depicting *PDCD4* mRNA levels in seven glioma cell lines vs. normal astrocytes. **(E)** Colony formation in glioma cells transfected with scrambled or *PDCD4* shRNAs. **(F)** Anchorage-independent growth in glioma cells transfected with scrambled or *PDCD4* shRNAs. *P < 0.05; **P < 0.01.

To elucidate the intricate role of PDCD4 in cancer, we first investigated the mRNA levels of *PDCD4* in several cancer types from the TCGA database. Among the 16 cancer types (originating in the glial cells, thyroid, lung, liver, pancreas, head and neck, stomach, colon and rectum, urethra, bladder, ureters and renal pelvis, prostate, cervix, endometrium, and skin), *PDCD4* was significantly down-regulated in glioma (Figure 1A). Next, we tested the mRNA and protein levels of PDCD4 in a panel of well-characterized human glioma cell lines and normal astrocytes. The analysis indicated PDCD4 to be significantly down-regulated in seven glioma cell lines than in normal astrocytes (Figures 1B,D), consistent with previous studies (Gaur et al., 2011; Liwak et al., 2013). To determine whether PDCD4 contributes to tumor suppression in glioma cells, we depleted the expression levels of *PDCD4* using two different shRNAs (Figure 1C). We found that the knockdown of *PDCD4* suppressed colony formation in the monolayer culture, and anchorage-independent growth in soft agar assay (Figures 1E,F). Taken together, our data suggests PDCD4 to be down-regulated in glioma cells and acts as a tumor suppressor.

### AKT2, but Not AKT1 and AKT3, Interacted With PDCD4

**FIGURE 2:**
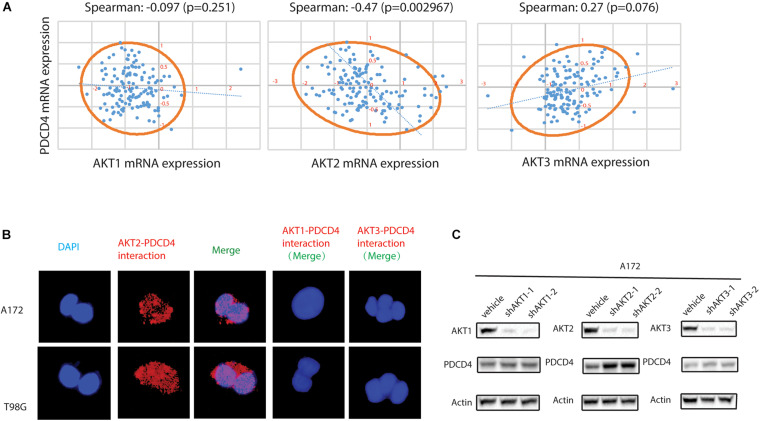
AKT2 interacts with PDCD4 in glioma cells. **(A)** The association of AKT1, AKT2, or AKT3 mRNA with PDCD4 mRNA. **(B)** Proximity ligation assay analysis indicating formation of AKT2/PDCD4 complex in glioma cells. **(C)** Western blot analysis for PDCD4 in glioma cells after knockdown of *AKT1*, *AKT2*, and *AKT3*.

Previous studies have showed that the down-regulation of PDCD4 was mediated via up-regulation of the protein kinase B (PKB/AKT) (Palamarchuk et al., 2005; Guo et al., 2011). However, it remains unclear which isoform of the AKT (AKT1, AKT2, or AKT3) regulates the expression of PDCD4. First, we analyzed the clinical glioma samples from the TCGA database and performed the Spearman’s rank correlation coefficient analysis using the XLSTAT software, and observed that the mRNA expression of *AKT2*, but not *AKT1* or *AKT3*, was inversely associated with that of *PDCD4* (Spearman = −0.47, *P* = 0.002967) (Figure 2A). Palamarchuk et al. (2005) reported that the AKT phosphorylates PDCD4 *in vitro* and *in vivo*, and Ozpolat et al. (2007) showed that AKT constitutively suppresses the expression of PDCD4 in acutemyeloid leukemia and breast cancer cells. To determine whether AKT2 interacts with and suppresses PDCD4, we performed the proximity ligation assay (PLA), which validated the formation of AKT2/PDCD4 complex in glioma cells, indicated using red dots in Figure 2B. Further, the western blot analyses in glioma cells with depleted levels of *AKT1*, *AKT2*, or *AKT3* showed that knockdown of *AKT2*, but not the *AKT1* or *AKT3* isoforms, resulted in increased expression of PDCD4 (Figure 2C). Taken together, these data suggest that AKT2, but not AKT1 or AKT3, interacts with and suppresses PDCD4 in the glioma cells.

### PDCD4 Regulates Expression of IL-5, CCL-5, VEGF, and CXCL10

**FIGURE 3:**
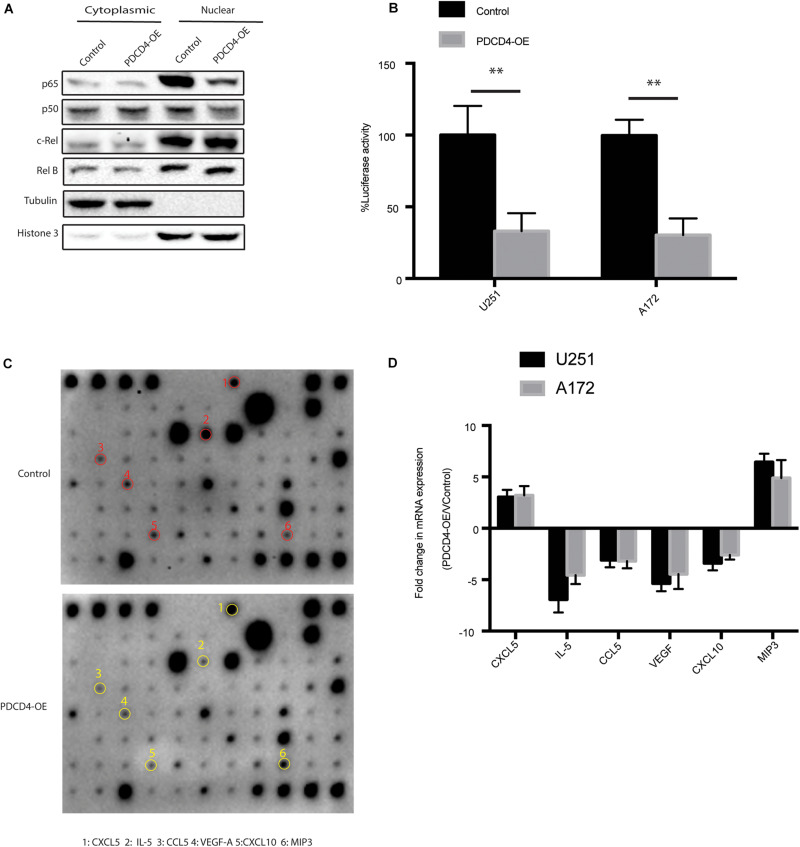
PDCD4 regulates production of IL-5, CCL-5, VEGF, and CXCL10. **(A)** Western blot analysis of nuclear and cytosolic fractions of indicated cells. Histone 3 and tubulin are used as nuclear protein marker and loading control, respectively. **(B)** Luciferase reporter assay showing NF-κB transcriptional activity in cells over-expressing *PDCD4*. **(C)** Cytokine assay indicating levels of cytokines and chemokines in conditional media collected from cells over-expressing *PDCD4*. **(D)** Real-time PCR analysis depicting mRNA fold changes of the indicated genes in the cytokine assay. **P < 0.01.

Previous studies showed that the expression of PDCD4 suppresses NF-kB transcriptional activation by inhibiting localization of p65 (Hwang et al., 2014; Su et al., 2017). To confirm whether the over-expression of *PDCD4* reduces the nuclear translocation of the NF-κB subunit, we fractionated the cell lysates over-expressing *PDCD4* into nuclear and cytoplasmic fractions, and then tested the subcellular localization of NF-κB subunits, *viz.* p65, p50, RelB, and c-Rel. The western blotting analysis showed that over-expression of *PDCD4* significantly reduced the levels of p65 and p50 proteins, but no significant change in that of RelB and c-Rel (Figure 3A). Furthermore, the luciferase reporter assay showed that the over-expression of *PDCD4* markedly reduced the transcriptional activity of NF-κB in the U251 and A172 cells (Figure 3B). Activation of the NF-κB is responsible for the transcriptional induction of pro-inflammatory cytokines, chemokines, and growth factors (Liu et al., 2017). Thus, to further explore which cytokines and chemokines could be suppressed by PDCD4, we collected the conditional medium from control cells or those over-expressing *PDCD4* for the cytokine assay. The data suggested that the levels of IL-5, CCL5, VEGF, and CXCL10 were reduced in the conditional medium from cells over-expressing *PDCD4*; however, the abundance of CXCL5 and MIP3 was enhanced by over-expression of *PDCD4* (Figure 3C). Moreover, these changes were confirmed using real-time PCR analysis in U251 and A172 cells stably over-expressing *PDCD4* (Figure 3D). Taken together, our analysis indicates that PDCD4 regulates the levels of IL-5, CCL-5, VEGF, and CXCL10.

### Depletion of *PDCD4* Promotes Angiogenic Activity via the VEGF-STAT3 Pathway

**FIGURE 4:**
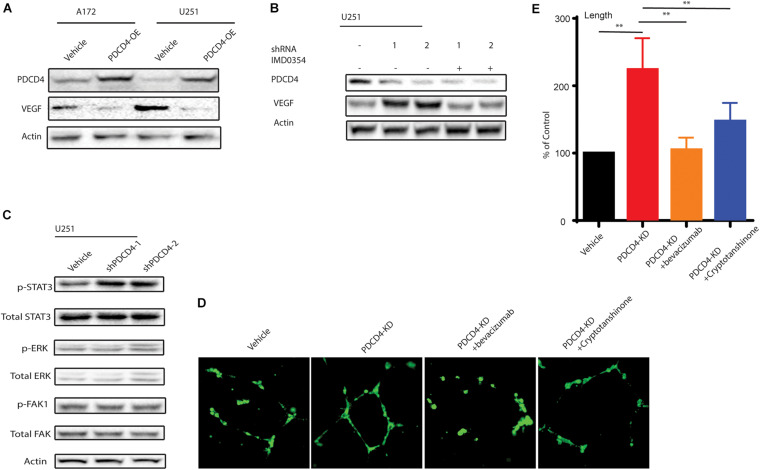
PDCD4 regulates angiogenesis via VEGF-STAT3 pathway. **(A)** Western blot analysis showing VEGF levels in cells over-expressing *PDCD4*. **(B)** Western blot analysis indicating VEGF expression *PDCD4*-depleted cells treated under indicated conditions. **(C)** Western blot analysis depicting p-STAT3 levels in *PDCD4*-knockdown cells. **(D)** Representative annotated images showing areas of tubes, loops, and branching points in HUVEC cells cultured with the indicated cells. **(E)** Quantification of length of tubes formed in response to indicated treatment. **P < 0.01.

Among many of these affected cytokines/chemokines, VEGF is the key mediator of angiogenesis, which is essential for development and growth of cancer (Shibuya, 2011). Since PDCD4 regulates the levels of VEGF, we hypothesized that PDCD4 may be involved in angiogenesis. First, we confirmed that over-expression of *PDCD4* inhibited the expression of VEGF using western blot analysis in the U251 and A172 cell lines (Figure 4A). To examine whether PDCD4 regulates VEGF in an NF-κB-dependent manner, we treated the *PDCD4*-depleted cells with IMD-0354 that selectively inhibits IKK activity in the NF-kB pathway (Tanaka et al., 2005). Western blot analysis showed that inhibition of NF-kB activity rescued the expression of VEGF in cells with depleted levels of *PDCD4* (Figure 4B). This confirmed that induction of VEGF after knockdown of *PDCD4* is dependent on the NF-kB pathway. Furthermore, VEGF stimulates the activation of diverse signaling proteins, including the focal adhesion kinase (FAK) (Chen et al., 2012), extracellular signal-regulated kinase (ERK) (Narasimhan et al., 2009), and members of the signal transducer and activator of transcription family (STAT) (Bartoli et al., 2000). The western blot analysis indicated that knockdown of *PDCD4* in glioma cells enhanced p-STAT3 levels, but not p-ERK or p-FAK1 (Figure 4C). To further explore the role of PDCD4 in angiogenesis, we collected conditional medium from *PDCD4*-depleted or control cells to culture the HUVEC cells for 48 h. We observed that knockdown of *PDCD4* resulted in thicker walls of cells and more tubes, (Figures 4D,E) indicating increased formation of tube-like structures. However, blocking the activity of VEGF or STAT3 with bevacizumab or cryptotanshinone, respectively, in *PDCD4*-depleted cells completely suppressed the formation of tube-like structures in HUVECs (Figures 4D,E). Taken together, these data indicate that depletion of *PDCD4* promotes angiogenic activity via the VEGF-STAT3 pathway.

### Over-Expression of *PDCD4* Impedes Glioma Tumorigenesis *in vivo*

**FIGURE 5:**
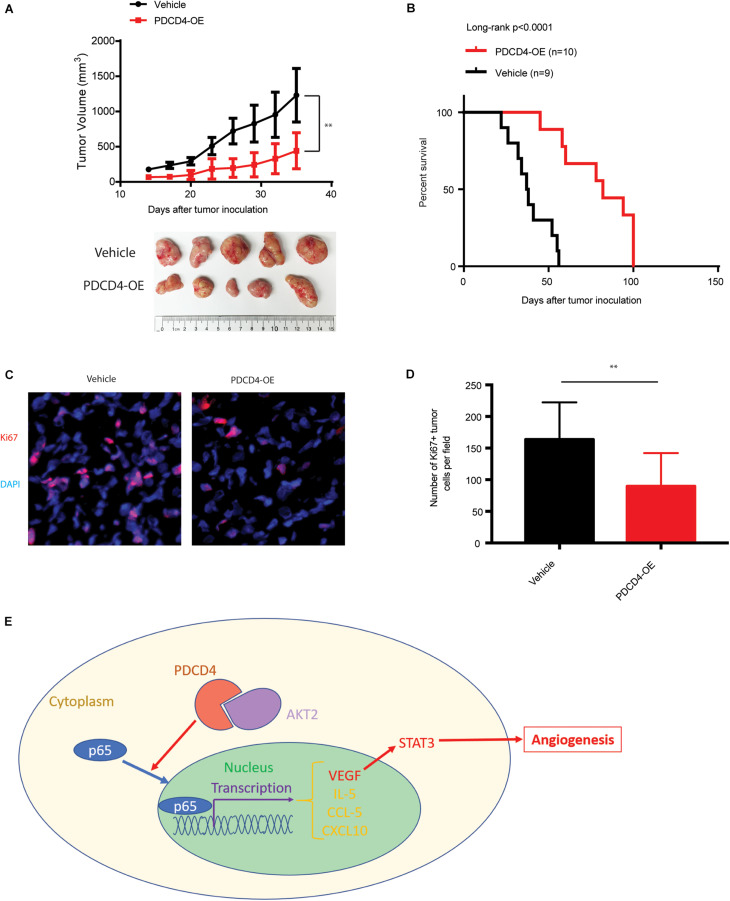
Over-expression of *PDCD4* impedes glioma tumorigenesis *in vivo*. **(A)** Growth kinetics of glioma cells stably over-expressing *PDCD4* following subcutaneous inoculation in nude mice. **(B)** Kaplan-Meier survival curves of glioma cells stably over-expressing *PDCD4* following subcutaneous inoculation in nude mice. **(C)** Representative immunofluorescence staining images of the indicated glioma tumors. **(D)** Quantification of Ki67 + proliferating cells in the indicated tumors. **(E)** Schematic of the proposed model depicting the binding of AKT2 and PDCD4 that regulates the nuclear translocation of p65, and thus leads to cytokine expression and angiogenic activity. **P < 0.01.

We next tested whether over-expression of *PDCD4* can suppress glioma growth *in vivo* using a xenograft BALB/c nude mice tumor model. Parent U251 cells or U251 cells over-expressing *PDCD4* were subcutaneously injected into the bilateral armpits of the nude mice. Consistent with our findings for anchorage-independent growth, the over-expression of *PDCD4* significantly suppressed the tumorigenic potential of glioma cells and prolonged the survival of tumor-bearing mice (Figures 5A,B). Moreover, immunofluorescence staining showed that *PDCD4*-over-expressing tumors had less proliferative cells (Ki67 + cells) (Figures 5C,D). Taken together, our data suggested that targeting PDCD4 by over-expression can block tumorigenesis and prolong overall survival duration, thus highlighting the need to develop potential drugs for clinical trials with PDCD4 in the future.

## Discussion

Glioma, the most lethal brain tumor occurring in adults, is characterized by abrupt growth, high invasiveness, and poor patient prognosis (Guillamo et al., 2001). Due to the high incidence and mortality rate among primary brain tumors, its treatment has remained largely unsuccessful (Shergalis et al., 2018). Thus, the identification of newer mechanism-based therapeutic strategies to improve survival duration is an important agenda in glioma. PDCD4 is ubiquitously expressed in normal tissues, with highest levels observed in the liver (Wang et al., 2013). However, PDCD4 is down-regulated in tumors, indicating it to be a potential anticancer target. Accumulating evidence has shown that PDCD4 is regulated by multiple mechanisms at the levels of transcription, translation, protein degradation, and post-translational modifications (Kroczynska et al., 2012). Dysregulation of PDCD4 alters the levels of several proteins involved in cell cycle, tumor progression, apoptosis, and differentiation in tumor cells (Jansen et al., 2005). Therefore, PDCD4 could be considered as a cancer therapy target.

In this study, we observed that the mRNA and protein levels of PDCD4 were significantly down-regulated in a panel of well-characterized human glioma cell lines, consistent with the TCGA data analysis. Down-regulation of *PDCD4* has been associated with cell proliferation. Depletion of *PDCD4* suppressed colony formation and anchorage-independent growth *in vitro*. While PDCD4 is reported to be less expressed in glioma, the underlying mechanism remained unclear. Here, we present evidence that AKT2, but not AKT1 or AKT3, interacts with PDCD4 leading to its suppression in glioma cells, and is consistent with the inverse relationship between mRNA levels of *AKT2* and *PDCD4* observed in the TCGA data analysis (Figure 5E).

Several recent studies have shown that PDCD4 is involved in the induction of inflammation. The *PDCD4*-depleted mice developed spontaneous tumors, mostly B-lymphoma, and showed significantly shorter life spans than their wild-type siblings, although they were resistant to the induction of inflammatory diseases, such as autoimmune encephalomyelitis and diabetes. Merline et al. reported that decorin regulates the inflammation and tumor growth via the PDCD4-microRNA-21 axis. A previous report showed that PDCD4 can significantly inhibit NF-κB activity, thus leading to the suppression of two NF-κB target genes. We showed that over-expression of *PDCD4* significantly reduced the p65 and p50 protein levels, but no obvious changes in levels of RelB and c-Rel in the nucleus, and then reduced the transcriptional activity of NF-κB. Moreover, we found that PDCD4 regulates the expression of IL-5, CCL-5, VEGF, and CXCL10 in glioma cells, which provides a therapeutic opportunity to block these cytokines (Figure 5E).

Tumor growth and metastasis depend on the angiogenesis triggered by chemical signals from tumor cells in the phase of rapid cell proliferation. The major mediator of tumor angiogenesis is VEGF, whose expression was found to be reduced upon over-expression of PDCD4 in the glioma cells. Whereas, the knockdown of *PDCD4* resulted in increased formation of tube-like structures. Furthermore, blocking VEGF or STAT3 in *PDCD4*-depleted cells rescued the formation of tube-like structures. Taken together, our data supports the role of PDCD4 in regulation of tumor angiogenesis.

It has been reported that the up-regulation of *PDCD4* sensitizes tumor cells to anti-tumor drugs, such as gemcitabine, cisplatin, tamoxifen, and geldanamycin. Our data indicate the potential clinical value of treatment with VEGF or STAT3 inhibitors in glioma patients expressing low levels of PDCD4. Moreover, PDCD4 is an important factor in angiogenesis besides its role in inflammation and cancer prevention (Figure 5E). Additionally, our *in vivo* data suggested that enhancing expression of *PDCD4* can block tumorigenesis and prolong overall survival, supporting the necessity to develop potential drugs for up-regulating PDCD4 in glioma patients in the clinics.

## Data Availability Statement

The original contributions presented in the study are included in the article/supplementary material, further inquiries can be directed to the corresponding author.

## Ethics Statement

The animal study was reviewed and approved by the Ethics Committee and Clinical Research Board at the Affiliated Hospital of Qingdao University.

## Author Contributions

GP and YY designed experiments in this study. GP, LH, ZC, and KX performed the research and acquired the data. GP, FY, LW, LS, LZ, HK, YW, and LY analyzed the data. GP, FY, QY, and YY interpreted the data. GP and YY wrote the manuscript and revised it. All authors gave the final approve of the version of to be published.

## Conflict of Interest

The reviewer ZW declared a shared affiliation with several of the authors, LY and QY, to the handling editor at the time of review.
